# Update on the Non-Pharmacological Management of Stroke Prevention in Patients with Atrial Fibrillation

**DOI:** 10.3390/jcm7020032

**Published:** 2018-02-12

**Authors:** Aneesh Tolat, Neal Lippman

**Affiliations:** Hoffman Heart and Vascular Institute, St. Francis Hospital and Medical Center, University of Connecticut School of Medicine, Hartford, CT 06105, USA; nlippman@ctheartbeat.com

**Keywords:** stroke, left atrial appendage, closure device, atrial fibrillation

## Abstract

Non-surgical left atrial appendage occlusion has emerged as an alternative to anticoagulant therapy in the management of stroke risk in patients with atrial fibrillation. This review reports on some of the more common devices that are currently being used to manage patients in this challenging group.

## 1. Introduction

Atrial fibrillation (AF) is recognized as the most common supraventricular arrhythmia, and it is known to be a major factor contributing to cardiovascular morbidity and mortality [1]. Among its most serious complications is the associated risk of embolic events, and in particular, embolic cerebrovascular accidents [2]. Accordingly, reduction of the risk of AF-associated stroke is a crucial component in the management of this arrhythmia.

The primary modality for stroke prevention in AF patients has been systemic anticoagulation, accomplished for many years with warfarin, and more recently with the introduction of the non-vitamin-K antagonist oral anticoagulants (NOACs), including dabigatran, rivaroxaban, apixaban, and edoxaban. While systemic anticoagulation provides effective stroke risk reduction in AF patients, it is subject to several drawbacks, including inconvenience and cost associated with daily medication administration, the need for frequent blood testing and dietary restrictions in patients treated with warfarin, the increased risk of bleeding complications, and the difficulty in management of anticoagulants in the setting of elective or non-elective invasive/surgical procedures or trauma. In addition, therapeutic anticoagulation with warfarin or NOACs preclude the use of intravenous thrombolysis in the case of acute ischemic stroke.

As a result of these limitations, mechanical alternatives to anticoagulation for stroke risk reduction have significant appeal and, as such, have been in development for more than 20 years, culminating in approval of the first such device, Boston Scientific’s Watchman, by the US Food and Drug Administration in 2015 [3]. Under the assumption that AF-related stroke results mostly from cardioemboli, and with the recognition that more than 90% of left atrial thrombi originate from the left atrial appendage (LAA) in patients with nonvalvular AF [4], mechanical occlusion of the left atrial appendage (LAAO) would be expected to prevent the formation of such thrombi and thereby prevent cardioembolic stroke.

This article will review the development and current status of LAAO as a therapy for stroke reduction in patients with AF.

## 2. Imaging for Assessment of Left Atrial Appendage Anatomy and Left Atrial Appendage Occlusion

Proper implantation of left atrial appendage occlusion devices requires pre-procedural assessment of appendage anatomy to guide device selection and deployment. In particular, the size of the appendage’s orifice is used to select the proper device size.

Various modalities can be used to assess LAA size and shape, including transesophageal echocardiogram (TEE), cardiac computed tomography (CCT) [5,6,7,8], and intracardiac echocardiography. CCT is preferred in the determination of LAA morphology and exclusion of pre-existing LAA thrombi due to its high spatial resolution and high negative predictive value for LAA thrombus [9,10].

Using CCT, Wang et al [5] described four major anatomic variations: Windsock (46.7% of patients), in which there is a single major lobe without significant bend; Cauliflower (29.1%), in which the LAA body is of short length prior to branching into multiple lobes; Chicken Wing (18.3%), in which there is a single obvious bend; and Cactus (5.9%), in which a major central lobe gives rise to multiple secondary lobes. Importantly, the LAA morphology may influence the risk of stroke in patients with AF, with the chicken wing morphology being associated with the lowest stroke rates [11]. In addition, Wang described the relationship of the LAA to the left superior pulmonary vein (LSPV), classifying this relationship into three categories: High type (30.2% of patients), with the LAA superior to the LSPV; Mid type (58.1%), with the LAA parallel to the LSPV, and Low type (11.7), where the LAA orifice is inferior to the LSPV. The LAA orifice was characterized as oval (68.9% of patients), foot-like (10%), triangular (7.7%), water-drop-like (7.7%), and round (5.7%). It was also noted that the LAA orifice diameter was best measured from the perimeter of the ostium for sizing of the occlusion device. In contrast to these findings, however, Su et al noted that the LAA orifice was oval in all 31 patients subject to gross anatomical examination [12]. Further, the LSPV, mitral valve, and left anterior descending coronary artery were also noted to be in close proximity to the LAA orifice, raising concern regarding potential injury to these structures during LAAO device deployment. The risk of injury to adjacent structures may be dependent on the specific occlusion device utilized [13].

TEE has also been utilized for pre-procedural assessment of LAA anatomy and for guidance during the implantation procedure. Both multiplane 2D imaging and real-time 3D imaging (RT3D) have been employed. Post-processing of RT3D images can directly measure the LAA orifice area without relying on geometrical assumptions [14] and is more accurate than 2D imaging alone [15]. However, multiplanar computed tomography (CT) imaging may be a more accurate method for pre-procedural assessment of LAA morphology and orifice area [16].

Utilizing the anatomic information gained from multiplanar CCT imaging with 3D image reconstruction, an algorithm for proper sizing and deployment of the Watchman device has been proposed [6]. Using this algorithm, Wang and colleagues demonstrated a low incidence of incorrect device sizing requiring placement of a second device. Interestingly, 3D printing of patients' left atrium and LAA was used in cases of ambiguous LAA orifice morphology to guide proper device selection and to allow ex-vivo testing of device deployment prior to the implantation procedure. CT image reconstruction was also helpful in correlation with intra-procedural TEE images in guiding device positioning. Specific measurements made via pre-procedure and/or intraprocedural imaging depend on the device to be employed, and they are used to guide device selection and size [17].

Intraprocedural imaging for device deployment is most commonly performed using TEE, which allows for visualization of the left atrium and LAA. Biplane TEE in the 45-degree and 135-degree views provides the most useful images during device deployment, although imaging in views from 0 through 180 degrees is recommended initially to fully assess LAA anatomy. Using TEE, the device position can be evaluated and Doppler interrogation used to assess for para-device leaks. Intracardiac echocardiography (ICE) can also be used for imaging during device implantation, and it has the advantage of potentially eliminating the need for general anesthesia during the procedure as is typically used to allow for TEE imaging [16]. While imaging of the intra-atrial septum for transseptal puncture can be easily accomplished, ICE imaging of the LAA is generally more challenging as compared to TEE [17]. Improved imaging of the LAA can be accomplished by advancing the ICE catheter transeptally into the LA but may require a second transseptal puncture for this to be accomplished.

Fluoroscopic imaging continues to be required during the device implantation procedure itself, during catheter placement, transseptal catheterization, and device deployment. In addition, a pigtail catheter is advanced into the LAA itself to allow cineangiography of the LAA to confirm anatomy and dimensions. Views optimizing measurements of different portions of the LAA have been described [17]. Very experienced operators may reduce or eliminate the use of fluoroscopy for parts of the procedure such as transseptal puncture which can be performed utilizing only echocardiographic guidance.

Post-procedural imaging is generally carried out using TEE or more commonly CCT for assessment of LAAO device position and peri-device leaks. Post-procedure CT scanning may be superior to post-procedure TEE in assessing for peri-device leaks, some of which may be missed on TEE due to off axis imaging [6,7].

## 3. Left Atrial Occlusion Devices

The first device developed for LAAO was the Percutaneous Left Atrial Appendage Transcatheter Occlusion device, or PLAATO (Appriva Medical, Sunnyvale, CA, USA) [18]. This device consisted of a self-expanding nitinol cage in a range of diameters from 15 to 32 mm covered with a polytetrafluoroethylene (PTFE) membrane laminated to the cage structure. The membrane is intended to provide both occlusion of the LAA orifice and to support tissue ingrowth. Small anchors along the struts of the nitinol cage engage the LAA tissue to help with device anchoring. The PLAATO was shown to have a high rate of implantation procedural success and to reduce stroke or transient ischemic attack compared with the expected rate based on the CHADS_2_ score of the patient population in a multicenter registry of 64 patients [19]. The development of this device demonstrated the feasibility of LAAO for stroke prevention, but the device is no longer available for use.

**Figure 1:**
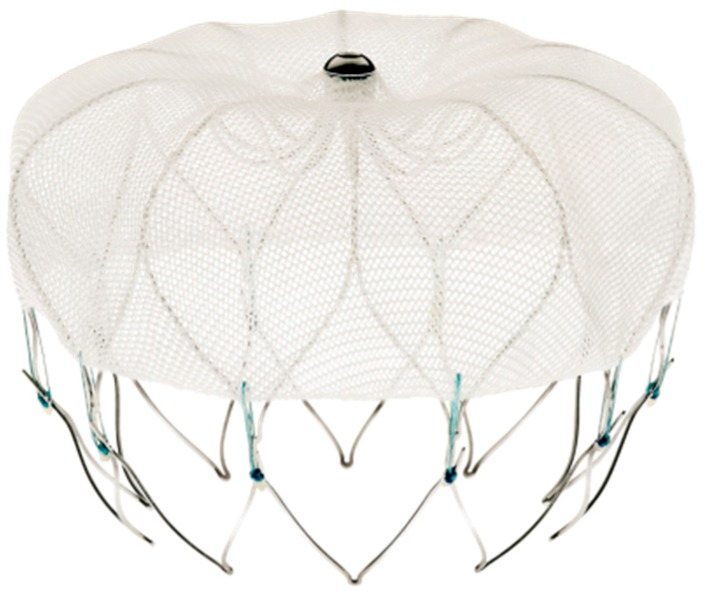
The Watchman device, with a 160-micron membrane on top, and the anchors are seen on bottom. (Image reproduced with permission from Boston Scientific.)

The Watchman device (Boston Scientific, Marlborough MA, USA), seen in Figure 1, also consists of a nitinol frame covered with polyester fabric, and it is also placed in the LAA via transseptal catheter delivery. The polyester fabric covers the proximal 50% of the device depth and is designed to prevent thrombi from embolizing out of the LAA. The device is available in 6 sizes from 16 to 30 mm. The Watchman is presently the only LAAO device approved for use in the United States by the Food and Drug Administration (FDA).

The LARIAT device (SentreHEART, Palo Alto, CA USA) utilizes an epicardial snaring technique with a pre-tied suture that is used to lasso the LAA and thus occlude the LAA [20,21]. Placement of the LARIAT requires both percutaneous transseptal access to the left atrium and transcutaneous pericardial access. A magnetically tipped guide wire is positioned in the LAA via the transseptal puncture, and the lasso is placed via the pericardial approach using the guide wire to aid in positioning the lasso device. The LARIAT is 510(k) US FDA approved for soft tissue approximation and/or ligation with a pre-tied polyester suture, but not specifically for LAAO.

**Figure 2:**
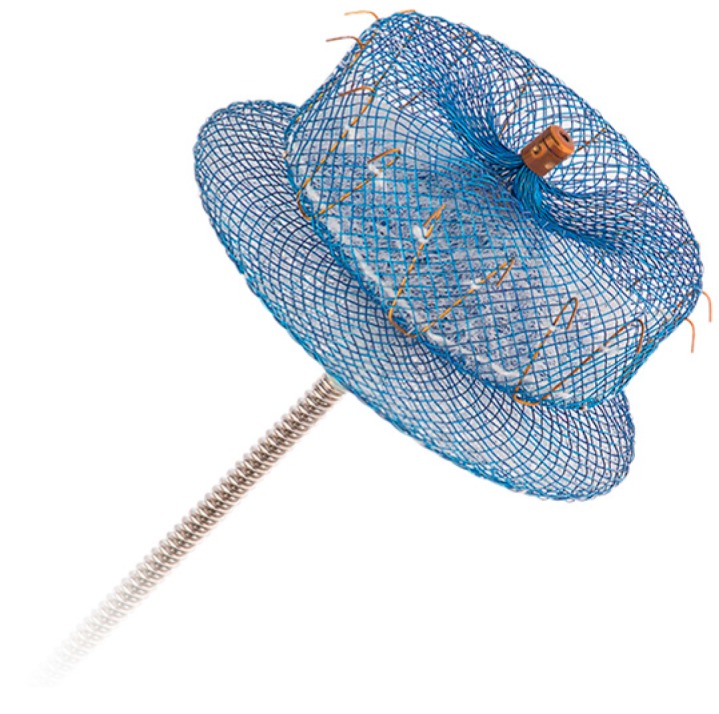
The Amplatzer Amulet device (reproduced with permission from Abbott).

The Amplatzer Cardiac Plug (St. Jude Medical), seen in Figure 2, is a self-expanding nitinol mesh made up of two components, a distal lobe and a more proximal disk-shaped component, which are connected and both covered with polyester [22]. The distal lobe provides anchoring of the device in the LAAO while the proximal disk covers the LAA orifice. In contrast to the Watchman device, the point of occlusion with the Amplatzer is on the left atrial side of the appendage rather than within the LAA. A second generation device, the Amplatzer Amulet [23], was released in 2013 and incorporates a larger sized disk component and a recessed screw on the disk. The device is delivered via a transseptal catheter approach.

Several other LAAO devices are in earlier stages of development, but none have reached the level of experience and testing of the above devices.

## 4. Clinical Trial Data

There have been several pivotal trials evaluating the efficacy and safety of LAAO devices as compared to warfarin anticoagulation. These studies evaluated LAAO in patients with long term contraindications to warfarin anticoagulation. As mentioned in the earlier section, the Watchman device is the only one with FDA approval and has been the most extensively studied. The first randomized trial evaluating the Watchman device was the PROTECT AF (Watchman Left Atrial Appendage System for Embolic Protection in Patients with Atrial Fibrillation) study reported by Holmes [24] and colleagues. The study randomized 707 patients with CHADS Score ≥1 to either warfarin or Watchman device implantation. The composite primary endpoint consisted of stroke, systemic embolism, and cardiovascular death. The trial endpoint of noninferiority was met with 3.0 events per 100 patient years in the Watchman group as compared to 4.3 in the warfarin group. However, when assessing the safety endpoints of life threatening bleeding and significant bleeding as well as procedure related complications, there was a significantly higher risk (7.7%) of procedural complications including air embolism and pericardial effusion in the Watchman group as compared to that of the warfarin group. As experience with the procedure grew, procedure related complications declined as analyzed in a follow-up registry to the PROTECT AF study [25].

In the follow-up PREVAIL study [26] (Evaluation of the Watchman LAA Closure Device in Patients with Atrial Fibrillation Versus Long Term Warfarin Therapy), patients with a CHADS score of ≥2 or 1 with one additional risk factor were randomized in a 2:1 fashion to device implant or continuing warfarin therapy, respectively. At 18 months, the trial did not meet the first prespecified endpoint of noninferiority (stroke, systemic embolism, or cardiovascular death/unexplained death) when compared to warfarin. However, the second endpoint of stroke or systemic embolism >7 days post randomization met noninferiority. Finally, the safety endpoint was analyzed as all 7 day procedural complications occurred in 4.5% of Watchman patients in PREVAIL as compared to 8.7% in PROTECT AF.

A subsequent meta-analysis [27] of the PROTECT AF and PREVAIL trials was conducted by Reddy and colleagues in patients who were followed for 5 years. Both trials together enrolled 1114 patients for a total of 4343 patient-years. The composite endpoint of stroke, systemic embolism, or cardiovascular/unexplained death was similar between Watchman and warfarin. There was a trend of ischemic stroke/systemic embolism that was higher in the Watchman group, but did not reach statistical significance (Hazard Ratio (HR) 1.71; *p* = 0.08). However, most importantly, hemorrhagic stroke, disabling/fatal stroke, cardiovascular/unexplained death, all causes death, and post procedure bleeding favored the Watchman group (HR 0.2; *p* = 0.0022).

More recently, there have been studies evaluating the possibility of limiting or implanting the Watchman without post procedural anticoagulation. The recently reported 1-year outcome data from the Ewolution trial [28], showed that the ischemic stroke rate after Watchman was 1.1% in a large cohort of patients (73%) who were not taking anticoagulation post procedure. In addition, the Assessment of the Watchman Device in Patients Unsuitable for Oral Anticoagulation Trial ASAP TOO [29] was started in 2017 to evaluate, in a randomized way, whether patients at risk for stroke but not candidates for anticoagulation can have the Watchman device implanted and randomized to Watchman and aspirin/clopidogrel vs. single antiplatelet therapy or no therapy.

Only data in the form of registries exist on the other available forms of LAA occlusion. Other than the Watchman device, there have not been any randomized studies published on other devices used for LAAO. The Amplatzer cardiac plug and its subsequent version, the Amulet, have been approved and available in Europe, but there have not been any published randomized studies on its safety and effectiveness. Registry based studies have been published including the Amplatzer Cardiac Plug Registry [30] which showed in a subset of patients with previous intracranial bleeding that peri-procedural adverse event rates were low, and that the annual stroke rate post device implant was also low at 1.4%. A second generation of this device, the Amulet, is currently being evaluated in clinical trials.

As mentioned earlier, the LARIAT occlusion device is not approved for use as a LAAO device in the US. It requires both endocardial and epicardial access for LAAO which is performed using a snare technique. The device benefits from a theoretical advantage over other devices in that it does not require anticoagulation after implant since the snare is epicardial and is not in the blood pool. However, as demonstrated in multiple small series of patients, leaks are common, and anticoagulation is usually recommended. Two studies from 2014 have shown that the LARIAT device can be used successfully to occlude the LAA, but with elevated risk for complications. In the study published in Heart Rhythm 2014 by Miller et al. [31], acute success in closure of the LAA was accomplished in 93%, but 2% of patients had a transient ischemic attack (TIA) and 20% had pericardial effusions requiring drainage. An additional 9% had LAA perforation, of which two required surgical correction. A larger study published in JACC 2014 [32] showed a 9.7% major complication rate of death, myocardial infarction (MI), stroke, and cardiac surgery. Further studies are awaited on this device to help determine its role in LAA closure.

There are several other devices which are being evaluated to determine their suitability for closing the LAA. The Coherex Wavecrest device was approved for use in Europe, but is no longer available [33]. Several others such as the Lambre and Aegis devices are currently undergoing evaluation.

## 5. Conclusions

Non-pharmacologic treatment of atrial fibrillation associated stroke risk has now been evaluated and shown to be feasible, effective, and safe in patients who have a relative contraindication to long term warfarin anticoagulation. The Watchman device is the only device that has been evaluated in large randomized trials. Ongoing randomized studies are evaluating whether patients can be treated with LAAO and antiplatelet therapy without short term anticoagulation post implant. Multiple other technologies are currently being evaluated to determine whether they also can be used safely and effectively.

While LAAO is an important step forward in treating patients with AF in respect to stroke risk, it should also be noted that not all strokes occur from the LAA. In addition, LAAO has not been compared to NOACs, which have a more favorable adverse event profile as compared to that of warfarin. The concept of LAAO has dramatically evolved from a surgical ligation procedure, to a percutaneous procedure that continues to evolve. The role of LAAO in the care of the AF patient remains to be defined.

## References

[1] Lip G.Y., Tse H.F. (2007). Management of atrial fibrillation. Lancet.

[2] Wolf P.A., Abbott R.D., Kannel W.B. (1991). Atrial fibrillation as an independent risk factor for stroke: The Framingham Study. Stroke.

[3] (2015). FDA Premarket Approval. https://www.accessdata.fda.gov/cdrh_docs/pdf13/p130013a.pdf.

[4] Blackshear J.L., Odell J.A. (1996). Appendage obliteration to reduce stroke in cardiac surgical patients with atrial fibrillation. Ann. Thorac. Surg..

[5] Wang Y., deBiase L., Horton R.P., Nguyen T., Morhanty P., Natale A. (2010). Left atrial appendage studied by computed tomography to help planning for appendage closure device placement. J. Cardiovasc. Elec..

[6] Wang D.D., Eng M., Kupsky D., Myers E., Forbes M., Rahman M., Zaidan M., Parikh S., Wyman J., Pantelic M. (2016). Application of 3-dimensional computed tomographic image guidance to WATCHMAN implantation and impact on early operator learning curve: Single-center experience. JACC Cardiovasc. Interv..

[7] Saw J., Lopes J.P., Reisman M., McLaughlin P., Nicolau S., Bezerra H.G. (2016). Cardiac computed tomography angiography for left atrial appendage closure. Can. J. Cardiol..

[8] Ismail T.F., Panikkar S., Markides V., Foran J.P., Padley S., Rubens M.B., Wong T., Nicol E. (2015). CT imaging for left atrial appendage closure: A review and pictorial essay. J. Cardiovasc. Comput. Tomogr..

[9] Kim Y.Y., Klein A.L., Halliburton S.S., Popovic Z.B., Kuzmiak S.A., Sola S., Garcia M.J., Schoenhagen P., Natale A., Desai M.Y. (2007). Left atrial appendage filling defects identified by multidetector computed tomography in patients undergoing radiofrequency pulmonary vein antral isolation: A comparison with transesophageal echocardiography. Am. Heart J..

[10] Romera J., Husain S.A., Kelesidis I., Sanz J., Medina H.M., Garcia M.J. (2013). Detection of left atrial appendage thrombus by cardiac computed tomography in patients with atrial fibrillation: A metanalysis. Circ. Cardiovasc. Imaging.

[11] Di Biase L., Santangeli P., Anselmino M., Mohanty P., Salvetti I., Gili S., Horton R., Sanchez J.E., Bai R., Mohanty S. (2012). Does the left atrial appendage morphology correlate with the risk of stroke in patients with atrial fibrillation? Results from a multicenter study. J. Am. Coll. Cardiol..

[12] Su P., McCarthy K.P., Ho S.Y. (2008). Occluding the left atrial appendage: Anatomical considerations. Heart.

[13] Kar S., Hou D., Jones R., Werner D., Swanson L., Tischler B., Stein K., Huibregtse B., Ladich E., Kutys R. (2014). Impact of Watchman and Amplatzer devices on left atrial appendage adjacent structures and healing response in a canine model. JACC Cardiovasc. Interven..

[14] Perk G., Biner S., Kronzon I., Saric M., Chinitz L., Thompson K., Shiota T., Hussani A., Lang R., Siegel R. (2012). Catheter-based left atrial appendage occlusion procedure: Role of echocardiography. Eur. Heart J..

[15] Shah S.J., Bardo D.M.E., Sugeng L., Weinert L., Lodato J.A., Knight B.P., Lopez J.J., Lang R.M. (2008). Real-time three-dimensional transesophageal echocardiography of the left atrial appendage: Initial experience in the clinical setting. J. Am. Soc. Echocardiog..

[16] Chow D.H.F., Bieliauskas G., Sawaya F.J., Millan-Iturbe O., Kofoed K.F., Søndergaard L., de Backer O. (2017). A comparative study of different imaging modalities for successful percutaneous left atrial appendage closure. Open Heart.

[17] Saw J., Lempereur M. (2014). Percutaneous left atrial appendage closure: Procedureal techniques and outcomes. JACC Cardiovasc. Interven..

[18] Sievert H., Lesh M.D., Trepels T., Omran H., Bartorelli A., Della Bella P., Nakai T., Reisman M., diMario C., Block P. (2002). Percutaneous left atrial appendage transcatheter occlusion to prevent stroke in high-risk patients with atrial fibrillation: Early clinical experience. Circulation.

[19] Block P.C., Burstein S., Casale P.N., Kramer P.H., Teirstein P., Williams D.O., Reisman M. (2009). Percutaneous left atrial appendage occlusion for patients in atrial fibrillation suboptimal for warfarin therapy: 5-year results of the PLAATO (Percutaneous Left Atrial Appendage Transcatheter Occlusion) study. JACC Cardiovasc. Interv..

[20] Bartus K., Bednarek J., Myc J., Kapelak B., Sadowski J., Lelakowski J., Yakubov S.J., Lee R.J. (2011). Feasibility of closed-chest ligation of the left atrial appendage in humans. Heart Rhythm.

[21] Bartus K., Han F.T., Bednarek J., Myc J., Kapelak B., Sadowski J., Lelakowski J., Bartus S., Yakubov S.J., Lee R.J. (2013). Percutaneous left atrial appendage suture ligation using the LARIAT device in patients with atrial fibrillation: Initial clinical experience. J. Am. Coll. Cardiol..

[22] Park J.W., Bethencourt A., Sievert H., Santoro G., Meier B., Walsh K., Lopez-Minguez J.R., Meerkin D., Valdés M., Ormerod O. (2011). Left atrial appendage closure with Amplatzer cardiac plug in atrial fibrillation: Initial European experience. Catheter. Cardiovasc. Interv..

[23] Kleinecke C., Park J.W., Gödde M., Zintl K., Schnupp S., Brachmann J. (2017). Twelve-month follow-up of left atrial appendage occlusion with Amplatzer Amulet. Cardiol. J..

[24] Holmes D.R., Reddy V.Y., Turi Z.G., Doshi S.K., Sievert H., Buchbinder M., Mullin C.M., Sick P., PROTECT AF Investigators (2009). Percutaneous closure of the left atrial appendage versus warfarin therapy for prevention of stroke in patients with atrial fibrillation: A randomised non-inferiority trial. Lancet.

[25] Reddy V., Holmes D., Doshi S.K., Neuzil P., Kar S. (2011). Safety of percutaneous left atrial appendage closure: Results from the Watchman left atrial appendage system for embolic Protection in Patients with AF (PROTECT AF) clinical trial and the Continued Access Registry. Circulation.

[26] Holmes D.R., Kar S., Price M.J., Whisenant B., Sievert H., Doshi S.K., Huber K., Reddy V.Y. (2014). Prospective randomized evaluation of the Watchman left atrial appendage closure device in patients with atrial fibrillation versus long-term warfarin therapy: The PREVAIL trial. J. Am. Coll. Cardiol..

[27] Reddy V.Y., Doshi S.K., Kar S., Gibson D.N., Price M.J., Huber K., Horton R.P., Buchbinder M., Neuzil P., Gordon N.T. (2017). 5-year outcomes after left atrial appendage closure: From the PREVAIL and PROTECT AF Trials. J. Am. Coll. Cardiol..

[28] Boersma L.V., Ince H., Kische S., Pokushalov E., Schmitz T., Schmidt B., Gori T., Meincke F., Protopopov A.V., Betts T. (2017). Efficacy and Safety of left atrial appendage closure with watchman with or without contraindication to anticoagulation: 1-year follow-up outcome data of the EWOLUTION trial. Heart Rhythm.

[29] Holmes D.R., Reddy V.Y., Buchbinder M., Stein K., Elletson M., Bergmann M.W., Schmidt B., Saw J. (2017). The assessment of the watchman device in patients unsuitable for oral anticoagulation trial. Am. Heart. J..

[30] Tzikas A., Freixa X., Llull L., Gafoor S., Shakir S., Omran H., Giannakoulas G., Berti S., Santoro G., Kefer J. (2017). Patients with intracranial bleeding and atrial fibrillation treated with left atrial appendage occlusion: Results from the Amplatzer Cardiac Plug registry. Int. J. Cardiol..

[31] Miller M.A., Gangireddy S.R., Doshi S.K., Aryana A., Koruth J.S., Sennhauser S., d’Avila A., Dukkipati S.R., Neuzil P., Reddy V.Y. (2014). Multicenter study on acute and long-term safety and efficacy of percutaneous left atrial appendage closure using an epicardial suture snaring device. Heart Rhythm.

[32] DiBiase L., Burkhardt J.D., Gibson D.N., Natale A. (2014). 2D and 3D TEE evaluation of an early reopening of the LARIAT epicardial left atrial appendage closure device. Heart Rhythm.

[33] Bergmann M.W. (2017). LAA occluder device for stroke prevention: Data on WATCHMAN and other LAA occluders. Trends Cardiovasc. Med..

